# Thresholding of the Elliott-Yafet spin-flip scattering in multi-sublattice magnets by the respective exchange energies

**DOI:** 10.1038/s41598-021-81177-9

**Published:** 2021-01-21

**Authors:** Artur Born, Régis Decker, Robby Büchner, Robert Haverkamp, Kari Ruotsalainen, Karl Bauer, Annette Pietzsch, Alexander Föhlisch

**Affiliations:** 1 Institute for Methods and Instrumentation for Synchrotron Radiation Research FG-ISRR, Helmholtz-Zentrum Berlin für Materialien und Energie, Albert-Einstein-Strasse 15, 12489 Berlin, Germany; 2 Institut für Physik und Astronomie, Universität Potsdam, Karl-Liebknecht-Strasse 24-25, 14476 Potsdam, Germany

**Keywords:** Magnetic properties and materials, Electronic properties and materials

## Abstract

How different microscopic mechanisms of ultrafast spin dynamics coexist and interplay is not only relevant for the development of spintronics but also for the thorough description of physical systems out-of-equilibrium. In pure crystalline ferromagnets, one of the main microscopic mechanism of spin relaxation is the electron-phonon (el-ph) driven spin-flip, or Elliott-Yafet, scattering. Unexpectedly, recent experiments with ferro- and ferrimagnetic alloys have shown different dynamics for the different sublattices. These distinct sublattice dynamics are contradictory to the Elliott-Yafet scenario. In order to rationalize this discrepancy, it has been proposed that the intra- and intersublattice exchange interaction energies must be considered in the microscopic demagnetization mechanism, too. Here, using a temperature-dependent x-ray emission spectroscopy (XES) method, we address experimentally the element specific el-ph angular momentum transfer rates, responsible for the spin-flips in the respective (sub)lattices of Fe$_{20}$Ni$_{80}$, Fe$_{50}$Ni$_{50}$ and pure nickel single crystals. We establish how the deduced rate evolution with the temperature is linked to the exchange coupling constants reported for different alloy stoichiometries and how sublattice exchange energies threshold the related el-ph spin-flip channels. Thus, these results evidence that the Elliott-Yafet spin-flip scattering, thresholded by sublattice exchange energies, is the relevant microscopic process to describe sublattice dynamics in alloys and elemental magnetic systems.

## Introduction

The development of ultrafast laser pulses down to a few tens of femtoseconds has allowed the measurement of spin relaxation in the order of 50 to 300 fs for pure 3*d* ferromagnets (see refs.^1–5^ for pure nickel and refs.^6,7^ for pure iron). In addition to the obvious implications in terms of data storage speed and efficiency, from a more fundamental point of view, the ultrashort pump pulse creates transient non-adiabatic, out-of-equilibrium conditions with accessible observables. Built on the 2-temperature model to describe the ultrafast thermalization of the heated electron bath in the lattice subsystems after an ultrashort pulse^8^, a phenomenological 3-temperature model (3TM) has been developed for the ultrafast demagnetization, which considers the lattice, the electrons and the spins subsystems separately^9^. However, a proper model of the underlying microscopic processes of the macroscopic observable of ultrafast demagnetization is still under debate. In particular, this model must satisfy the angular momentum conservation of the system. The most accepted microscopic model, the microscopic-3TM, is based on the Elliott-Yafet type scenario, where the electron-phonon (el-ph) scattering-driven angular momentum transfer between the electrons and the lattice is associated with a probability of spin-flip, leading to the longitudinal spin relaxation of the system^2,10–12^.

More recently, ultrafast pump-probe methods have been applied to magnetic alloys, where peculiar transient magnetic states were observed, like an ultrashort ferromagnetic alignment in a ferrimagnetic GdFe compound^13^. For the ferromagnetic alloys Fe$_{20}$Ni$_{80}$ and Fe$_{50}$Ni$_{50}$, first, the measured demagnetization time of nickel is faster than that of iron and second, the demagnetization of nickel in Fe$_{50}$Ni$_{50}$ happens faster than in Fe$_{20}$Ni$_{80}$^14–19^. These observations are in contradiction with the microscopic-3TM model, which considers the system as a single macrospin, and therefore, does not distinguish the different elements of the multisublattice. Moreover, even applying the microscopic-3TM to multisublattices, one would expect demagnetization time constants to be proportional to the ratio of the magnetic moments and the Curie temperature $\tau _{demag.} \propto \mu _{i}/T_C$, where $\mu _{i}$ is the atomic magnetic moment of the sublattice *i* in the alloy when taking the sublattices independently^2^. This again contradicts a faster time constant for nickel in Fe$_{50}$Ni$_{50}$ than in Fe$_{20}$Ni$_{80}$, since $\mu _{Ni}$ increases with the iron concentration with identical $T_C$ (see Table 1).

**Table 1:** Atomic magnetic moment, demagnetization time and Curie temperatures for Ni, Fe, Fe$_{20}$Ni$_{80}$ and Fe$_{50}$Ni$_{50}$.

	$\mu _{Ni}$ ($\mu _B$)	$\mu _{Fe}$ ($\mu _B$)	$\tau _{Ni}$ (fs)	$\tau _{Fe}$ (fs)	$T_C$ (K)
Ni	0.62	–	130 ± 40	–	629
Fe$_{20}$Ni$_{80}$	0.98 ± 0.04	2.31 ± 0.09	180 ± 40	300 ± 50	907
Fe$_{50}$Ni$_{50}$	1.18 ± 0.08	2.09 ± 0.08	80 ± 30	280 ± 50	804
Fe	–	2.2	–	222 ± 50	1044

Atomic magnetic moments are deduced from XMCD measurements at room temperature^16^. All demagnetization times, deduced from time resolved XMCD measurements at room temperature, are adapted from^14^. The Curie temperature for the pure elements are adapted from^20^ and from^14^ for the alloys.

In order to explain this apparent paradox in multisublattices, the intra- and intersublattice exchange interaction $J_{ij}$, where *i* and *j* denote the sublattice, have been proposed as an additional ingredient for the microscopic description of longitudinal spin dynamics^21,22^. The exchange as origin of the demagnetization of pure ferromagnets, i.e. not multisublattice, is not relevant since for Heisenberg exchange interactions, the total angular momentum is conserved. This means that in this case, $up\rightarrow down$ and $down\rightarrow up$ spin transitions must be equal and thus, the macrospin $S_z=1/N\sum _{i}s_{i_z}$ remains unchanged. In a multisublattice, like a FeNi alloy, spin-flips of one sublattice can be compensated by spin-flips in the other sublattice, that means $S_{z}(Ni)=1/N(Ni)\sum _{i}s_{i_z}(Ni)$ and $S_z(Fe)=1/N(Fe)\sum _{i}s_{i_z}(Fe)$ can change independently leaving the total macrospin $S_z$ constant. Despite the success of this model for the understanding of real systems like GdFe, direct experimental evidence of the role of the exchange interaction in the ultrafast demagnetization of multisublattice has been missing so far. One of the reasons is the difficulty to get access to the el-ph scattering responsible for the spin-flips independently of other processes like the exchange interaction, since ultrafast laser pump-probe techniques integrate over all mechanisms.

Here, we present the experimental determination of the el-ph scattering rates at the nickel and iron atoms in the two different alloys Fe$_{20}$Ni$_{80}$ and Fe$_{50}$Ni$_{50}$ as well as in pure nickel. Our method is based on the core-hole clock method, where the core-hole lifetime is used as a time reference to deduce the timescale of dynamic processes like the el-ph scattering timescale^23–30^. Following the stringent dipole selection rules, scattered electrons do not participate to the core-hole decay in the case of e.g. scattering-driven angular momentum transfer or spin-flip . Thus, in these cases, a higher scattering probability is accompanied by a reduction of the decay probability linked to a visible loss of emission peak intensity. According to the Bose-Einstein statistics, the temperature dependence allows a control over the phonon population and thus, over the el-ph scattering timescale. In comparison with the Elliott-Yafet scenario of ultrafast demagnetization, where the el-ph scattering responsible for the electron spin-flip occurs during an ultrashort transient time, in our method we rather place the system in a condition that mimics the transient non-adiabatic conditions after an ultrashort laser pulse, but in a static way.

## Results

To get access to an observable outcome of the el-ph scattering rate in the 3*d* bands, we created independently nickel and iron $2p_{3/2}$ core-holes with two incident energies between the $L_2$ and $L_3$ edges of nickel and iron, at 864 eV and 716 eV, respectively. These energies were chosen in order to minimize competing effects, like resonant excitations or Coster-Kronig decay. We acquired XES spectra including the peaks of the dipolar 3*s* $\rightarrow$ $2p_{3/2}$ core-to-core and the 3*d* $\rightarrow$ $2p_{3/2}$ valence-to-core hole radiative transitions. The el-ph scattering induces an angular momentum transfer, which, due to the dipole selection rules, leads to a lower probability of $3d\rightarrow 2p_{3/2}$ decay, visible as a reduction of the corresponding emission peak in XES spectra. Similarly to the case of pure nickel, we attribute the derived el-ph scattering driven angular momentum transfer to the spin-flip^23^.

**Figure 1:**
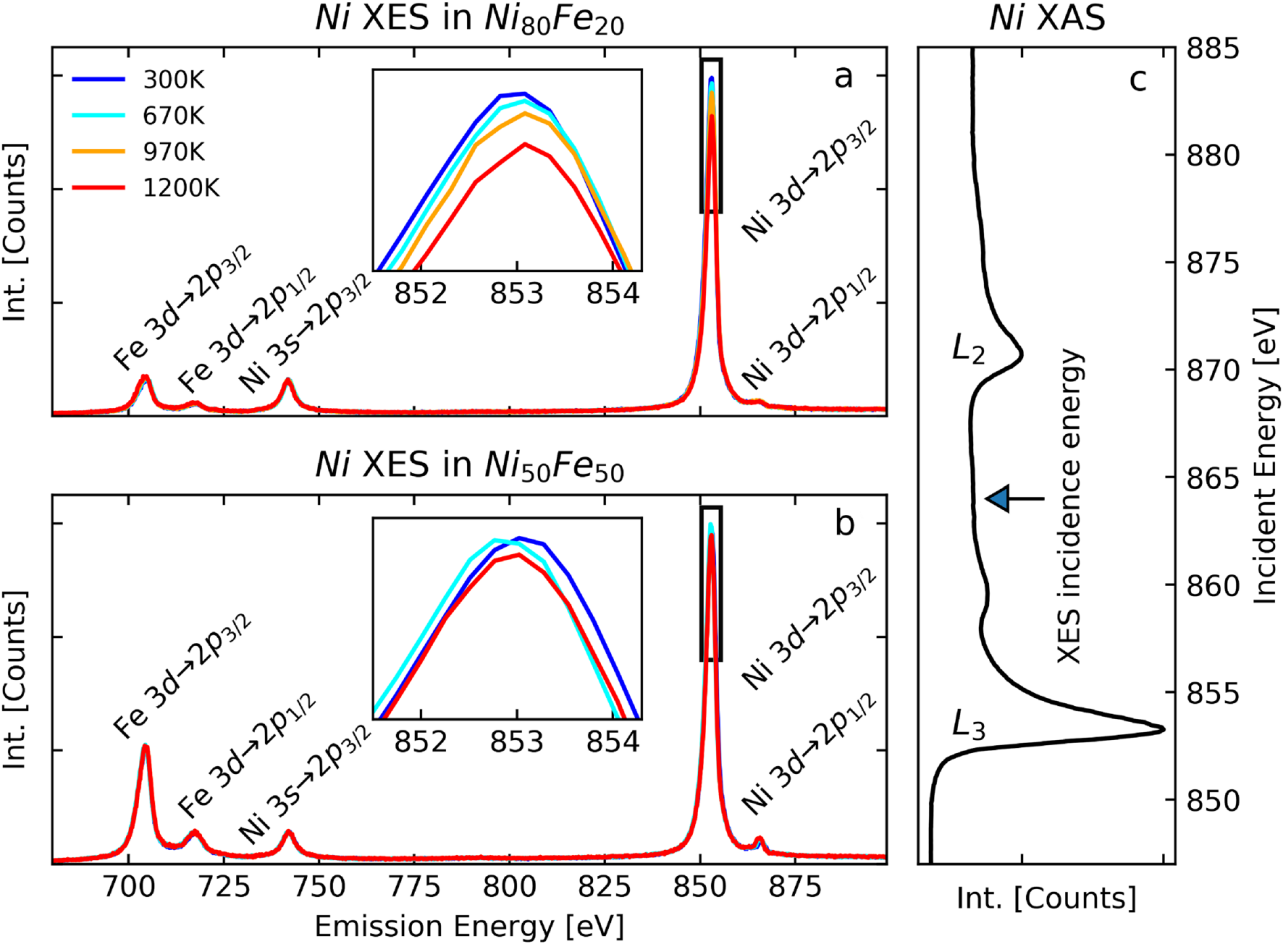
Temperature-dependent XES of nickel. Temperature dependent Ni *L*-edge XES spectra in Fe$_{20}$Ni$_{80}$ and Fe$_{50}$Ni$_{50}$ are shown in (**a**) and (**b**) respectively. The insets are zoom in of the $3d \rightarrow 2p_{3/2}$ peak. An increase of the temperature leads to a reduction of intensity of the $3d \rightarrow 2p_{3/2}$ peak. (**c**) XAS spectrum of nickel. The arrow indicates the photon energy used for the XES measurements.

**Figure 2:**
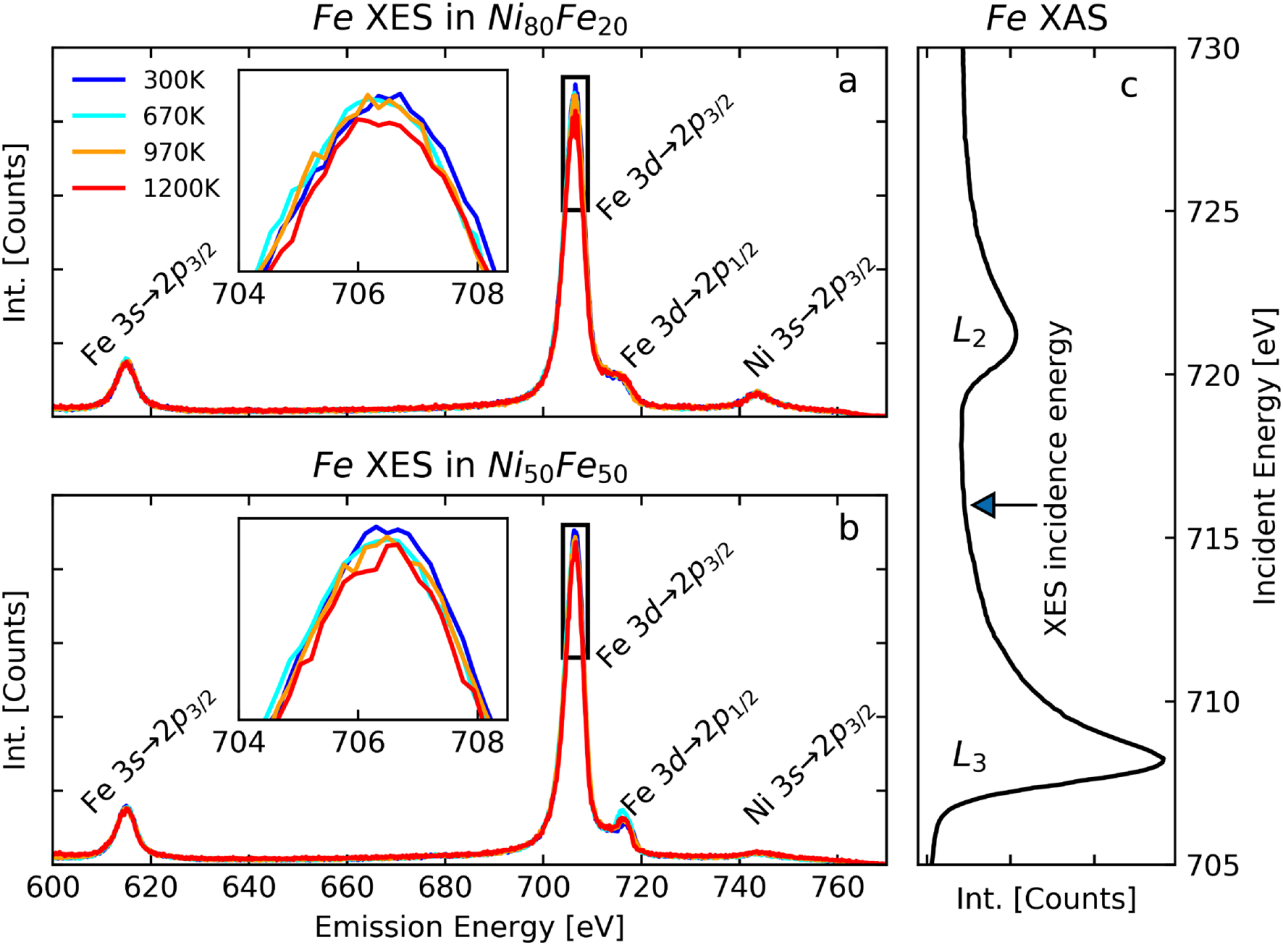
Temperature-dependent XES of iron. Temperature dependent Fe *L*-edge XES spectra in Fe$_{20}$Ni$_{80}$ and Fe$_{50}$Ni$_{50}$ are shown in (**a**) and (**b**) respectively. The inset are zoom in of the $3d \rightarrow 2p_{3/2}$ peak. An increase of the temperature leads to a reduction of intensity of the $3d \rightarrow 2p_{3/2}$ peak. (**c**) XAS spectrum of iron. The arrow indicates the photon energy used for the XES measurements.

The temperature-dependent XES spectra of nickel and iron in the two alloys Fe$_{20}$Ni$_{80}$ and Fe$_{50}$Ni$_{50}$ are shown in Figs. 1 and 2, respectively, together with the x-ray absorbtion spectroscopy (XAS) spectra. The energy scale of the XES spectra were calibrated using the tabulated values for pure metals^31^ and were normalized against the $3s\rightarrow 2p_{3/2}$ peak intensity. The latter is expected to be constant with the temperature, since no inelastic el-ph scattering can happen in the full 3*s* core level and thus, no change in the $3s\rightarrow 2p_{3/2}$ decay probability are possible (see^32^).

**Figure 3:**
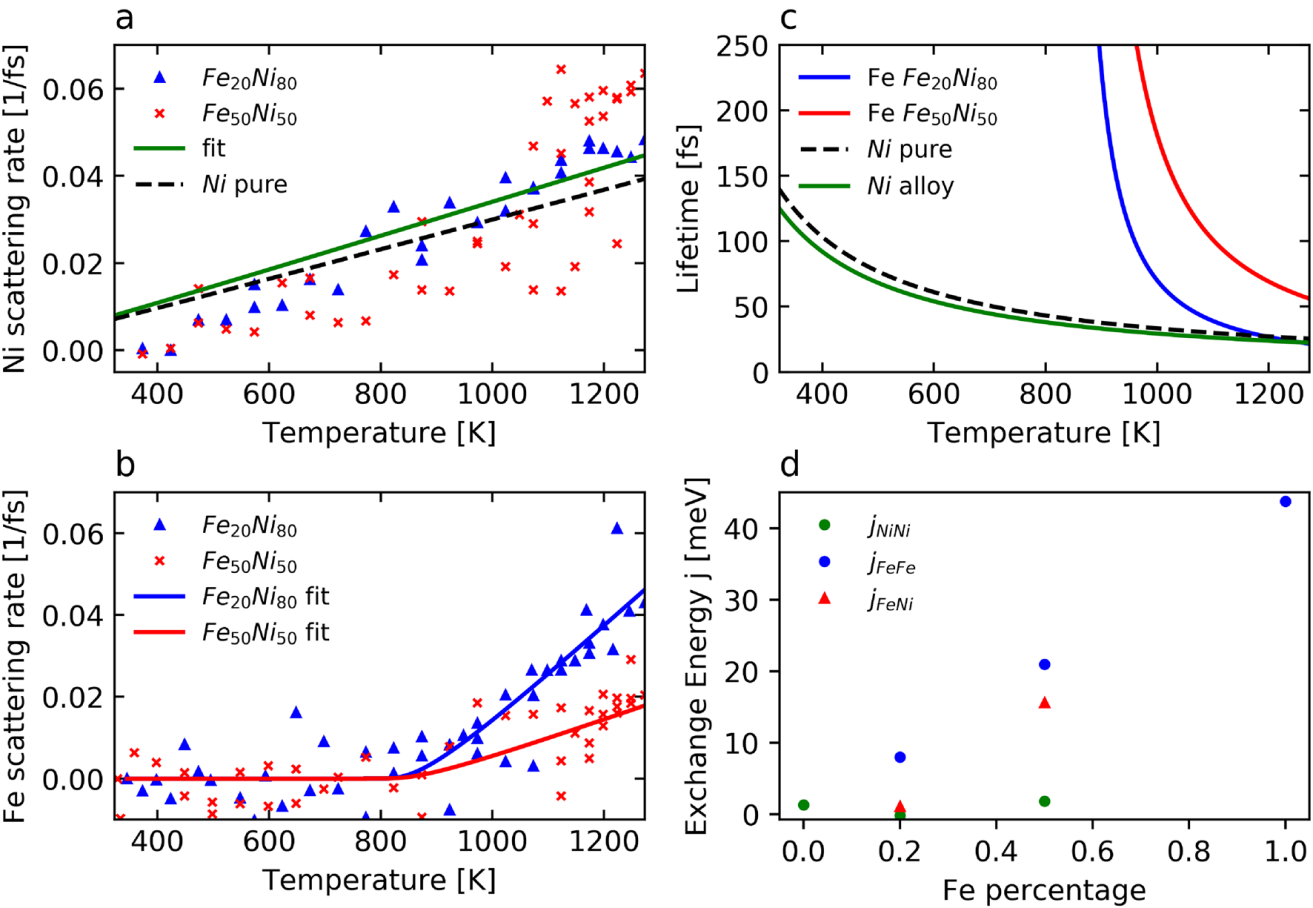
Scattering rates and exchange interaction energies. (**a**) and (**b**) Inelastic scattering rate in the different stoichiometries for nickel (**a**) and iron (**b**). The dashed line in (**a**) shows the rate for pure nickel^23^. The green line is the fit for both alloy stoichiometries. For clarity, the error bars are shown in the Supplementary Information only^32^. (**c**) Corresponding scattering lifetimes. (**d**) Intralattice exchange energy vs. the iron content. Values for the pure nickel and the Fe$_{20}$Ni$_{80}$ are adapted from ref.^35^. Values for pure Fe$_{50}$Ni$_{50}$ and pure iron are adapted from^16^.

To determine the el-ph scattering rate we analysed the $3d \rightarrow 2p_{3/2}$ peak area evolution with temperature of the normalized spectra. In both alloys we observe a reduction of the peak intensity when increasing the temperature for nickel and for iron. In the framework of the core-hole clock method, the el-ph scattering rate $R(T) = 1 / \tau _{el-ph}$ (where $\tau _{el-ph}$ is the inelastic el-ph scattering time) responsible of the angular momentum transfer is determined by considering that the ratio of the core-hole lifetime and the el-ph scattering time is equal to the ratio of the probabilities of decay with (incoherent) and without (coherent) scattering:

$$\begin{aligned} \frac{\tau _{core-hole}}{\tau _{el-ph}}=\frac{P_{scatt. + decay}}{P_{no~scatt. + decay}} \end{aligned}$$

or,

$$\begin{aligned} R(T)=\frac{1}{\tau _{core-hole}}\cdot \frac{A_{inc}}{A_{coh}}=\frac{1}{\tau _{core-hole}}\cdot \frac{A_{cold}-A_{hot}}{A_{cold}}, \end{aligned}$$

where $\tau _{core-hole}$ is the core-hole lifetime of the excited state, $P_{scatt. + decay}$ and $P_{no~scatt. + decay}$ are the probability of decay with and without prior scattering, respectively, $A_{coh}=A_{cold}$ is the peak area in the spectra, which is not affected by el-ph scattering and $A_{inc}=A_{cold}-A_{hot}$ is the temperature-dependent contribution. The derived rates *vs*. temperature for nickel and iron in both alloys are shown in Fig. 3a,b, respectively, where $\tau _{core-hole}= 1.88\,\text {fs}$ for nickel and $\tau _{core-hole}= 1.37\,\text {fs}$ for iron^33^. For clarity, uncertainties are not included in the figure. A corresponding figure with the error bars is shown in the Supplementary Information^32^.

We fitted our experimental data using the Bose-Einstein statistic and assuming a temperature dependent and a temperature independent component of the rate:

$$\begin{aligned} R(T)=C_{indep}+C_{dep}\cdot \frac{1}{e^{\frac{E_{ph}}{k_bT}}-1}=\frac{1}{\tau _{el\text{-}ph}}, \end{aligned}$$

where $C_{indep}$ describes the constant scattering rate and $C_{dep}$ is the factor for the temperature-dependent contribution. $C_{dep}$ can be understood as the probability of el-ph scattering and thus, of angular momentum transfer or spin-flip in the case of nickel and iron^23^. $E_{ph}$ is the mean phonon energy and is very similar for different stoichiometries. We used the reported mean value of the phonon density of states in the iron nickel alloys $E_{ph}=24$ meV measured with nuclear resonant inelastic x-ray scattering^34^. Following the Elliott-Yafet scenario, we assumed el-ph scattering as a driving mechanism for the spin relaxation. For nickel the Bose-Einstein statistics is sufficient to fit the data in the entire temperature range. However, for iron the Bose-Einstein statistics fits the experimental data for temperatures above 850 K only. Below 850 K the data are fitted by a constant, accordingly to the discussion presented below. The angular momentum transfer rate can be seen in terms of timescales, which is depicted in Fig. 3c.

## Discussion

In this section, we will discuss three remarkable features in the temperature dependant el-ph rates and timescales derived from our experiments: (1) the similar el-ph scattering rates for nickel in both alloys and pure nickel. (2) the difference in the rate for different FeNi compositions and (3) two regimes in the iron rate evolution with the temperature. We will show that the microscopic-3TM model is not sufficient to account for these three features and that the intra- and intersublattice exchange interactions in the different systems must be considered. Based on our observations we propose a model for the ultrafast demagnetization in multi-sublattice systems, which can be understood in terms of a threshold linked to the exchange coupling energy.

The first feature is the similarity of el-ph scattering rates and timescales for nickel for both alloys and for pure nickel^23^. As mentioned above, the microscopic-3TM predicts a demagnetization timescale, and thus, an el-ph scattering timescale proportional to $\mu _i/T_C$^2^. However, the significant increase of the nickel magnetic moment with the iron concentration, by e.g. almost a factor 2 between pure nickel and nickel in Fe$_{50}$Ni$_{50}$ (see Table 1), is not compensated by the increase of $T_C$, which is 629 K for pure nickel and around 800–900 K for the alloys^16,36^.

The second feature is the lower el-ph scattering rate for iron in Fe$_{50}$Ni$_{50}$ than in Fe$_{20}$Ni$_{80}$ (see Fig. 3a,b). This is also in contradiction with the microscopic-3TM model, which postulates a rate proportional to $T_C / \mu _{at}$. Indeed, since the iron atomic magnetic moment decreases when increasing the iron concentration with constant $T_C$ (see Table 1), we would expect a higher rate with a higher iron concentration^2,16^.

Similarly to the conclusions drawn from the measurements of ultrafast demagnetization, these two features demonstrate the need of the intra- or intersublattice exchange interaction as an additional ingredient in the microscopic mechanisms^21^. Reported values of the intrasublattice exchange interactions between the nickel atoms $J_{Ni-Ni}$ and the iron atoms $J_{Fe-Fe}$, and the intersublattice exchange interactions between the nickel and iron atoms $J_{Ni-Fe}$ for our three stoichiometries, are plotted in Fig. 3d. We note a striking correspondence between these values and our el-ph scattering rates and timescales. Indeed, $J_{Fe-Fe}$ and $J_{Ni-Fe}$ increase with the iron concentration and are both much higher than $J_{Ni-Ni}$: in Fe$_{20}$Ni$_{80}$, $J_{Fe-Fe}$ is about 8 meV, whereas $J_{Ni-Ni}$ and $J_{Ni-Fe}$ are only around 1 meV. In Fe$_{50}$Ni$_{50}$, $J_{Ni-Ni}$ remains rather unchanged, while $J_{Fe-Fe}$ increases further up to 23 meV. $J_{Ni-Fe}$ also dramatically increases up to 15 meV. The $J_{Ni-Fe}$, although smaller than $J_{Fe-Fe}$ by a factor of 71%, presents a comparably increasing trend with the iron concentration.

The evolution of $J_{Fe-Fe}$ and $J_{Ni-Fe}$ can be compared to the iron el-ph scattering rates, which is smaller for Fe$_{50}$Ni$_{50}$ than for Fe$_{20}$Ni$_{80}$. In other words, for higher exchange interaction more thermal energy is needed to reach a certain scattering rate. A similar reasoning can be made from the scattering timescale point of view shown in Fig. 3c. Furthermore, a constantly low $J_{Ni-Ni}$ (about 1 meV) is reported for all stoichiometries, which corresponds to the identical and relatively low el-ph scattering timescales for nickel in all cases, including pure nickel, in the sense that a relatively low thermal energy is needed to overcome the low $J_{Ni-Ni}$ and enable the el-ph scattering induced spin-flip.

These considerations are consistent with the third remarkable feature, which is the presence of two regimes in the evolution of the el-ph scattering rate in the alloys: a constant and zero rate below $\sim$ 850 K to an almost linear increase of the rate above this temperature (see Fig. 3b). Interestingly, this temperature corresponds closely to the $T_C$ of the alloys. Mentink et al. proposed the existence of three regimes defined by a temperature below, above and at $T_C$, where the longitudinal spin relaxation have an exchange, relativistic (where the dynamics is dominated by the transfer of angular momentum with the lattice) or both origins, respectively^21^. Our method gives access to the el-ph scattering-driven longitudinal relaxation, or relativistic regime, only, where $k_BT$ > *J*. Such a cross-over in the scattering rates of nickel is not apparent. The reason can be the lower $T_C$ (600 K) since $J_{Ni-Ni}$ is identically low for all systems and we are almost always in the $k_BT$ > *J* regime for nickel. Since the measured scattering rates of nickel are similar, the interlattice exchange interaction $J_{Ni-Fe}$ seem to play no role for nickel. Analogously, this indicates that the interlattice exchange interaction plays no role for the evolution of the scattering rate evolution of iron.

The general concept of out-of-equilibrium physical systems implies the idea of interplay *via* energy transfer and sequential dominance of the different subsystems for the total dynamics. Our static approach, where the electrons and lattice systems are placed in thermal equilibrium to measure the constant resulting spin-flip scattering rate, allows to disentangle this sequence by directly observing the occurrence of the dominant driving subsystem. Specifically, our results show that despite the thermal equilibrium of the electron and lattice subsystems, the electron-lattice interaction channel for the spin-flip scattering remains closed for a sublattice below a certain threshold temperature determined by the sublattice exchange coupling energy. This is visible here at e.g. 700 K, where this channel is open for the nickel sublattice, but closed for the iron sublattice. This means that below the temperature threshold, the properties of the sublattice are dominated by the electron-electron interactions *via* the exchange coupling. At the threshold temperature, the electron-lattice coupling channel opens up and the angular momentum transfer becomes the dominant process for the spin-flip scattering. Since the lattice temperature increases mainly the amount of the phonons and not $E_{ph}$, our results indicate that only the electron subsystem temperature can open the electron-lattice coupling channel.

Importantly, our experiments substantiate that only the electron thermal energy is relevant to overcome the exchange coupling energy barrier. The lattice temperature, which governs the amount of phonons, influences the probability of angular momentum transfer only if this channel is open. In the framework of ultrafast demagnetization, we know that during the thermalization of the electron system in the first 100 fs after the laser pulse, the sub-picosecond magnetic dynamics relies on the presence of the hot electrons subsystem that are not yet thermalized with the lattice subsystem, since the latter process occurs in a time frame of 1 ps^2,8^. Our results allow a better understanding of the microscopic process sequence of demagnetization. We demonstrate that, due to the very fast rise of the electron subsystem temperature, even for low lattice subsystem temperatures, the angular momentum transfer spin-flip channel can be open. More conceptually, one could suppose that different demagnetization mechanisms can also be thresholded, which would help understanding more recent results showing delayed demagnetization in metallic alloys for different components^17,18,37,38^.

## Conclusion

In conclusion, we determined the spin-flip rate originating from the angular momentum transfer rate driven by el-ph scattering at the nickel and iron atoms of FeNi alloys and pure nickel by temperature dependent and element-specific X-ray emission core-hole clock spectroscopy. In contrast to crystalline nickel, where the spin-flip occurs already at relatively low temperatures, the spin-flip of iron in alloys is triggered only above a temperature, close to T$_C$, which corresponds to the temperature needed to overcome the exchange interaction energy. Thus, we propose a phenomenological model, where the exchange interaction energy corresponds to a barrier, which has to be overcome by the electron thermal energy in order to enable the angular momentum transfer between electrons and lattice. In our particular experiment, this trigger temperature is low for nickel in all systems and corresponds to the low intrasublattice exchange interactions energy of nickel. This implies that the intersublattice exchange coupling does not play a role for the temperature threshold of nickel and suggests that similarly, the temperature threshold for the spin-flip scattering in general depends on the iron intrasublattice exchange energy only. Our results represent a further step in the understanding of ultrafast demagnetization and confirm the importance of the exchange interaction and the predicted temperature crossover between the exchange coupling and the angular momentum transfer as origin of the spin-flip. More generally, they show the combined roles of the microscopic mechanisms in non-equilibrium physics.

## Methods

The experiments were performed at the PETRA III P04 beamline and at the BESSY II U49-2_PGM-1 and UE52_SGM beamlines during the multi-bunch operation using the BESSY II SolidFlexRIXS end station. The element- and temperature-dependent XES spectra were acquired using Fe$_{20}$Ni$_{80}$ and Fe$_{50}$Ni$_{50}$ single crystals. The base pressure was in the low $10^{-8}$ mbar range but rose up to the $10^{-7}$ mbar range for the highest temperatures. The samples were placed on two different positions of the same sample holder for similar experimental conditions. A metal plate between the samples prevented possible material deposition from one sample to the other during the long acquisition times at high temperatures. The data acquisition was delayed until the system thermalized after each temperature change. Several series of measurements were performed, whereby the temperature was increased or decreased before acquisition, in order to rule out non-reversible structural changes. The XAS spectra were acquired with the same samples in total electron yield mode at room temperature. The pure nickel data are adapted from^23^.

## Supplementary Information

Supplementary Information.
