# Supplementary material for: Thresholding of the Elliott-Yafet spin-flip scattering in multi-sublattice magnets by the respective exchange energies
Source: Sci Rep. 2021 Jan 21;11:1883. doi: 10.1038/s41598-021-81177-9 (PMC7820427; doi:10.1038/s41598-021-81177-9)
Supplement: Supplementary file 1 — Supplementary Information. [file 41598_2021_81177_MOESM1_ESM.pdf]

Thresholding of the Elliot-Yafet spin-flip scattering in multi-sublattice magnets by the respective exchange energies

Artur Born[1,2], Régis Decker[1,*], Robby Büchner[1,2], Robert Haverkamp[1,2], Kari Ruotsalainen[1], Karl Bauer[1], Annette Pietzsch[2], and Alexander Föhlisch[1,2,*]

[1]Institute for Methods and Instrumentation for Synchrotron Radiation Research FG-ISRR, Helmholtz-Zentrum Berlin für Materialien und Energie Albert-Einstein-Strasse 15, 12489 Berlin, Germany
[2]Institut für Physik und Astronomie, Universität Potsdam, Karl-Liebknecht-Strasse 24-25, 14476 Potsdam, Germany
[*]regis.decker@helmholtz-berlin.de, alexander.foehlisch@helmholtz-berlin.de

Raw data

Raw temperature dependent L-edge XES spectra corresponding to the normalized data shown in FIG.1 and 2 of the main text are shown in FIG. 1. Each spectrum was accumulated for 20 min.

Data normalization is needed, due to thermal dilatation of the experimental setup. The spectrometer is finely aligned at room temperature. Most importantly, by heating the sample, several parts of the experimental setup, like the sample holder and the cryostat change their length and position due to thermal dilatation[1]. For example, we observed a change of a few millimeters for the cryostat length between room and the highest temperatures. Since the spectrometer has a very small entrance slit (several 100 μm), already the slightest misalignment will result in an overall photon count loss, visible in spectra. Such a misalignment also results in a slight peak shift of 10 meV between room and the highest temperatures. As discussed in the main text, we normalized the spectra by considering that fully occupied bands or levels do not allow inelastic scattering, because of the lack of free states for the electron to be scattered into. Therefore, the area of the $3s \rightarrow 2p_{3/2}$ decay peak was used for the spectra normalization.

Structural changes of FeNi

One of the characteristic feature visible in the scattering rate of iron of FIG.3b is the slope change, from zero to finite, of the rate *vs*. temperature occurring at 800 K. One way to interpret this change of slope is the crossover between the exchange coupling and the angular momentum transfer regimes for the spin-flip, as discussed in the main text. Another way of interpretation could be a structural phase transition. Indeed, the phase diagram of permalloy shows a structural change, which starts around 650 K. With higher temperature the concrete $FeNi_3$ fcc structure changes into a solid solution, γFe and γNi for $Fe_{50}Ni_{50}$ and $Fe_{20}Ni_{80}$, respectively. This is also connected with a change of the lattice parameter, which has been shown to modify the exchange energies[2]. Indeed, structural changes can lead to changes of the electronic band structure, mean phonon energy and exchange energies. Thus, in the evolution of the scattering rate for nickel we would expect to see a feature like a change of slope related to variations in electron or phonon structure occurring at the temperature corresponding to structural phase transitions. Instead, the nickel scattering rate in FeNi alloys behaves similar to the one of pure nickel, which is consistent with absence of change in the exchange energy. The fact that we do not see such a feature further supports our interpretation. Therefore, even considering a structural phase transition close to the crossover temperature, the problem might be again reduced, at least partly, to an exchange coupling to angular momentum transfer spin-flip crossover.

Data uncertainties

Fig. 2 shows the electron phonon spin-flip scattering rate for Ni and Fe in both measured alloys $Fe_{80}Ni_{20}$ and $Fe_{50}Ni_{50}$ including the error bars. The data analysis is explained in detail in the main text.

References

1. Decker, R. *et al.* Measuring the atomic spin-flip scattering rate by x-ray emission spectroscopy. *Sci. Reports* **9**, 8977 (2019).

2. Yu, P., Jin, X. F., Kudrnovský, J., Wang, D. S. & Bruno, P. Curie temperatures of fcc and bcc nickel and permalloy: Supercell and green's function methods. *Phys. Rev. B* **77**, 054431 (2008).

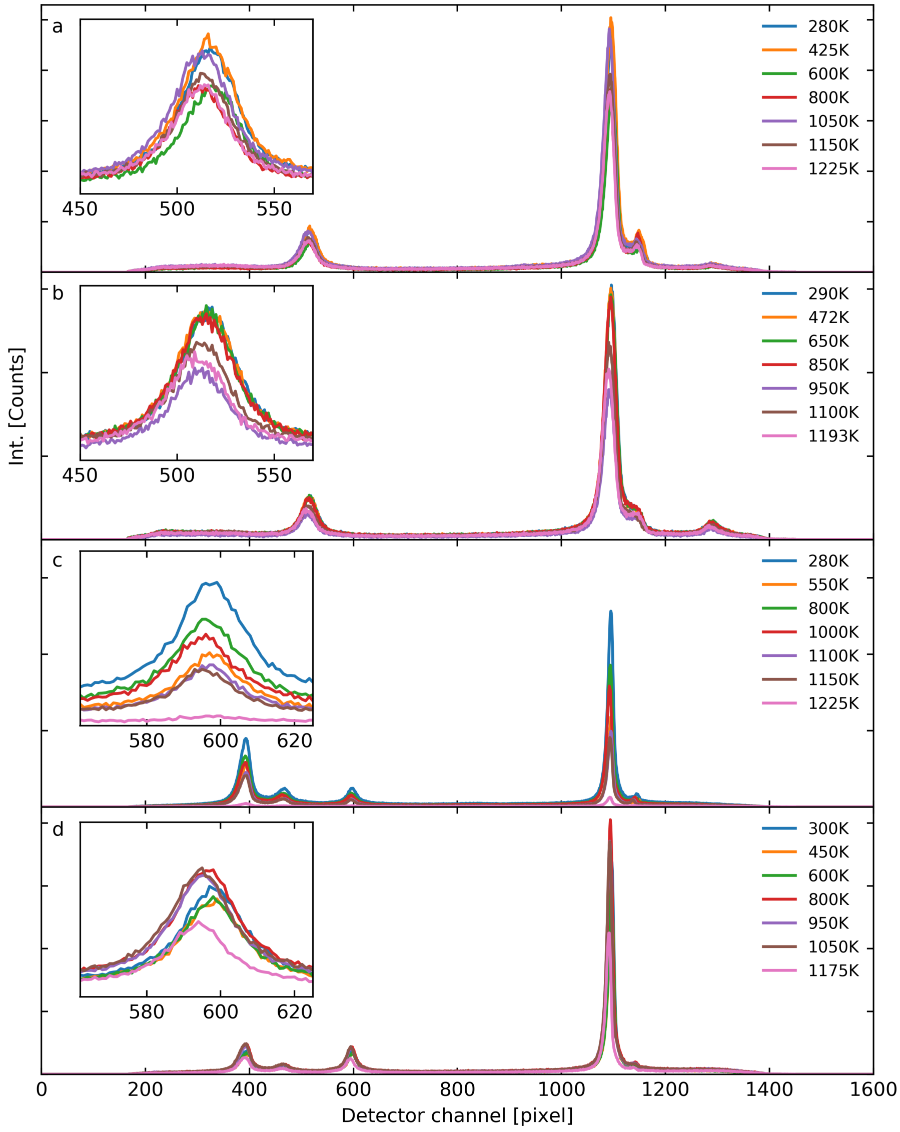

Figure 1. Temperature dependent L-edge XES raw spectra. a) and b) depict the Fe spectra for $Fe_{20}Ni_{80}$ and $Fe_{50}Ni_{50}$, respectively. c) and d) depict the Ni spectra for $Fe_{20}Ni_{80}$ and $Fe_{50}Ni_{50}$, respectively. The insert shows the $3d \rightarrow 3s$ peak.

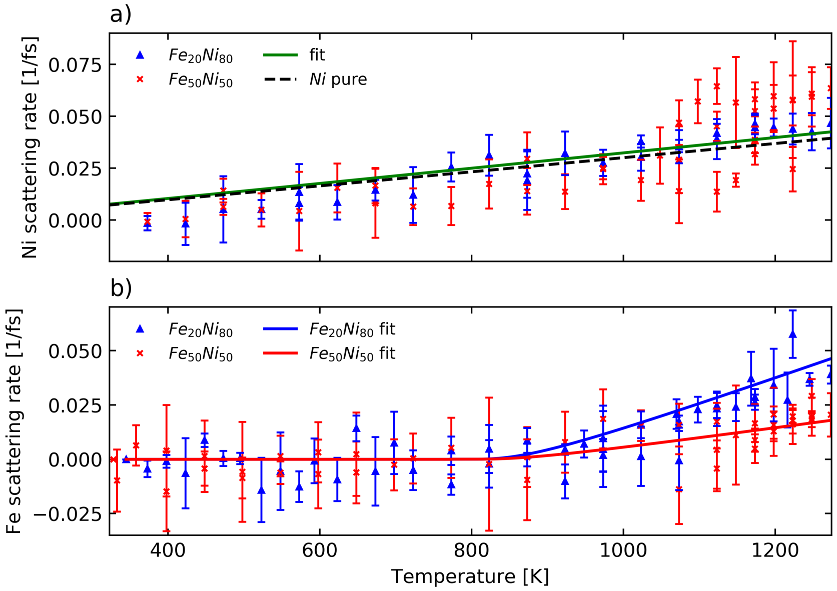

Figure 2. a) Nickel electron phonon spin-flip scattering rate in $Fe_{20}Ni_{80}$ (blue) and $Fe_{50}Ni_{50}$ (red) and the corresponding fit (green). Fit for the scattering rate in pure Nickel is depicted in black. b) Iron electron phonon spin-flip scattering rate in $Fe_{20}Ni_{80}$ (blue) and $Fe_{50}Ni_{50}$ (red) and the corresponding fits.
